# Editorial: The digital transformation of dental and maxillofacial practice towards preventive, personalised, and precision medicine

**DOI:** 10.3389/froh.2025.1611158

**Published:** 2025-05-09

**Authors:** Giorgio Lo Giudice, Ciro Emiliano Boschetti, Enrico Nastro Siniscalchi

**Affiliations:** ^1^ Department of Medicine and Surgery, University of Enna “Kore”, Enna, Italy; ^2^ Department of Biomedical and Dental Sciences and Morphofunctional Imaging, University Hospital “G. Martino”, University of Messina, Messina, Italy; ^3^ Multidisciplinary Department of Medical-Surgical and Dental Specialties, Oral and Maxillofacial Surgery Unit, University of Campania “Luigi Vanvitelli”, Naples, Italy

**Keywords:** maxillofacial surgery, dentistry, precision medicine, thee-dimensional printing, computer-assisted surgery, patient-specific implant, virtual surgical planning, computer-aided design

**Editorial on the Research Topic**
The digital transformation of dental and maxillofacial practice towards preventive, personalised, and precision medicine

## 1 Introduction

Dentistry and maxillofacial surgery are undergoing significant transformation, driven by an incessant push towards precision and personalized medicine. This approach aims to individualize diagnosis, treatment planning, and surgical execution, leveraging digital and advanced technologies and an increasingly detailed understanding of individual patient needs. The six recent publications analyzed here demonstrate this evolution, illustrating how various specialist areas are integrating innovations to improve accuracy, effectiveness, and clinical outcomes.

Perrotta et al. analyzed the effectiveness and duration of the “canine first” technique for impacted maxillary canines. A key finding was that an alpha angle of less than 22° served as a favorable prognostic indicator for faster eruption. The “canine first” technique, involving early surgical exposure, proved effective, with the alpha angle offering predictive value for treatment duration.

Krasovsky et al. presented a novel method for secondary 3D reconstruction of malunited zygomatic fractures using 3D planning and printing for re-fracture, precise repositioning, and fixation with surgical guides and patient-specific implants (PSIs), avoiding the more invasive coronal approach. Demonstrated in two cases, the approach yielded exceptional accuracy, highlighting its potential to mitigate human error in complex 3D procedures and its applicability to other secondary reconstructions.

Chaitanya et al. introduced “Nallan's Lines” as an innovative aid to enhance intraoral radiographic technique. This cross-over study assessed a gyroscopic LASER device projecting these lines as a reference for the bisecting angle technique. Results indicated statistically significant improvements in the accuracy of projected tooth dimensions and a reduction in common technical errors like apical cutting and horizontal overlap following training with Nallan's Lines, thereby reducing the necessity for retakes.

Rachmiel et al. described a cost-effective, in-house 3D technology-based method for the optimal positioning of standard lengthening devices for mandibular osteogenic distraction using in-house 3D designed and printed cutting guides derived from virtual surgical planning (VSP) to adapt them to the individual contour of the mandibles. Successful application in three patients with mandibular retrognathism demonstrated precise alignment of the adapted, resulting in excellent clinical outcomes thus showcasing a more accessible alternative to expensive custom-made distractors.

Lo Giudice et al. presented a clinical case showcasing the application of virtual surgery and 3D printing in managing a pathological mandibular fracture associated with medication-related osteonecrosis of the jaw (MRONJ). Virtual models derived from CT scans facilitated precise planning of sequestrectomy, debridement, and fracture reduction. The resulting mandibular model served as a template for preoperatively shaping a titanium reconstruction plate. Intraoperative and follow-up assessments confirmed the model's accuracy in reflecting postoperative mandibular dynamics and a reduction in surgical time, underscoring the potential of virtual surgery and 3D prototyping in complex MRONJ cases.

Babiuc et al. detailed a digitally guided tooth autotransplantation case utilizing surgical templates and a 3D-printed replica of the donor tooth. A maxillary premolar with fused roots was successfully transplanted to an edentulous mandibular site. Digital planning, root-specific surgical guides, and the 3D tooth replica ensured accuracy, a favorable prognosis, and reduced treatment duration. The donor tooth replica allowed for optimal preparation of the recipient site prior to extraction, minimizing root trauma and extraoral time.

These six articles, while addressing distinct clinical challenges, share a common thread: the integration of advanced technologies to enhance the precision and predictability of treatments in dentistry and maxillofacial surgery.

Perrotta et al. and Chaitanya et al. focus on refining diagnostic and treatment planning stages. Both aim to equip clinicians with more precise tools for informed decision-making and error reduction. Krasovsky et al., Rachmiel et al., and Lo Giudice et al. highlight the transformative impact of virtual surgical planning (VSP) and 3D printing in surgical interventions. These studies underscore the capacity of 3D technology to overcome limitations of traditional surgical techniques, offering enhanced personalization and accuracy. Babiuc et al.’s work explores the application of digital workflows in the highly precise procedures, exemplifying how digital technologies can improve predictability and efficacy in delicate dental surgical procedures.

A key observation across these studies is the central role of 3D technology driving precision medicine. It empowers clinicians to visualize, plan, and execute interventions with unprecedented detail and accuracy, emphasizing the need for greater accessibility and cost-effectiveness of these tools. A significant parallel focus is the consistent drive to reduce errors and enhance patient outcomes. Regardless of the specific intervention, be it shortening orthodontic treatment, restoring facial structures, improving radiography, guiding osteogenesis, managing complex pathologies, or optimizing transplants, the ultimate goal remains the delivery of more effective and personalized care.

The research highlighted in this editorial collectively provides a compelling snapshot of the evolving landscape of dentistry and maxillofacial surgery, moving towards increasingly precise and personalized medicine. The overarching aim of this evolving paradigm is to move beyond generalized protocols and embrace individualized approaches. The advancements showcased align with a global trend across various medical disciplines towards personalized healthcare where technologies like 3D imaging, virtual planning, and patient-specific devices, reflect a growing recognition that a “one-size-fits-all” approach often falls short in addressing the complexities of individual patients. Moreover, this Research Topic also explores ways of making these tools more accessible, thus broadening their potential impact on patient care.

The findings contribute to a growing body of evidence supporting the transformative potential of digital workflows and personalized approaches in achieving more successful and patient-centric outcomes in the fields of dentistry and maxillofacial surgery. As research continues to evolve and these technologies become more integrated into routine clinical practice, the promise of truly individualized and optimized patient care in these disciplines moves closer to realization.

